# Laparoscopic approach for Hartmann reversal procedures

**DOI:** 10.4103/0972-9941.28180

**Published:** 2006-12

**Authors:** H Scheidbach, H Lippert

**Affiliations:** Department of Surgery, Otto-von-Guericke-University Magdeburg, Leipziger Str. 44, D-39120 Magdeburg

Minimally invasive surgery has been one of the most important surgical innovations over the last 15 years. In 1990, not only was the first laparoscopic sigmoid resection described, but also the first laparoscopic anterior rectal resection, the first laparoscopic right hemicolectomy and the first laparoscopic stoma creation. Since then, laparoscopic colorectal surgery has found broad acceptance in the world.

The laparoscopic Hartmann reanastomosis procedure - first described in 1993 - however, only played a marginal role at that time. The international literature currently offers only a few publications, mostly case reports and small series with 10 to 20 patients. Thus, the results of a total of approximately 100 patients who underwent laparoscopic Hartman reanastomosis have been published by now.[1]

The small number of patients included in our own analyses emphasized the minor importance of laparoscopic reanastomosis after the Hartmann operation. The prospective multicenter study of the Laparoscopic Colorectal Surgery Study Group is one of the biggest worldwide registers for laparoscopically operated colorectal patients and includes more than 5000 patients in Central Europe who were registered between 1995 and 2005. Only 71 patients were included in this study who underwent a laparoscopic Hartmann reversal. This patient group represents only 1.4% of the total laparoscopic operations and therefore plays an unimportant role.

The morbidity for these cases was 16.9% and the mortality 1.4%; both aspects showed no significant complication rates. In contrast, a reoperation rate of 8.4% was found. The mean operating time is 164 min (range 60-410 min) and thus is longer than for elective sigmoid resections with primary anastomosis.[2]

The complication rate of 14.1% and the conversion rate of 12.7% were significantly increased, generally due to extensive intraabdominal adhesions. Since in Europe, the indication for laparoscopic reanastomosis after Hartmann operation is almost solely a sigmoid diverticulitis with peritonitis, strong adhesions have to be expected in such cases. Although a conversion to laparotomy is no primary complication, it may be important to mention that a conversion is associated with a significant poorer outcome. On the other hand, patient selection in order to avoid high conversion rates is almost impossible.

In the subsequent work, V. Golash completes the already available results with his experiences with 12 patients who underwent a laparoscopic Hartman reversal. Particularly with regard to mortality and conversion his results are comparable with those from the available literature and with our own experiences. He shows that this laparoscopic procedure is safe and efficient.

Despite the potential low postoperative morbidity and mortality, a short hospital stay, a reduced postoperative pain and an accelerated convalescence, this procedure is technically challenging and should therefore be reserved to experienced minimally invasive surgeons. Reason for that is a relatively long operating time as well as a significant higher conversion and intraoperative complication rate.

Regardless of the fact that specialized centers are able to achieve outstanding results with this procedure, we would like to emphasize that a laparoscopic Hartmann reversal currently cannot be recommended in general.
